# Oral Contraceptives Induce Time- and Intestinal Segment-Dependent Shifts in the Gut Microbiota

**DOI:** 10.3390/nu17162591

**Published:** 2025-08-09

**Authors:** Anna Clapp Organski, Anjali Reddivari, Lavanya Reddivari, Douglas K. Brubaker, Kelly N. Z. Fuller, John P. Thyfault, Tzu-Wen L. Cross

**Affiliations:** 1 Department of Nutrition Science, Purdue University, West Lafayette, IN 47907, USA; 2 Department of Electrical and Computer Engineering, Purdue University, West Lafayette, IN 47907, USA; 3 Department of Food Science, Purdue University, West Lafayette, IN 47907, USA; 4 Center for Global Health and Diseases, Department of Pathology, School of Medicine, Case Western Reserve University, Cleveland, OH 44106, USA; 5 The Blood Heart Lung Immunology Research Center, Case Western Reserve University and University Hospitals, Cleveland, OH 44106, USA; 6 Department of Cell Biology and Physiology, University of Kansas Medical Center, Kansas City, KS 66160, USA; 7 Kansas City Veterans Affairs Research Service, Kansas City Veterans Affairs Hospital, Kansas City, MO 64128, USA

**Keywords:** gut microbiota, oral contraceptives, estrogen, metabolic health

## Abstract

Oral contraceptives (OCs) containing estrogen and/or progesterone are the second most common form of female contraception in the United States. While endogenously synthesized estrogen is known to provide protective effects against cardiometabolic diseases, exogenous forms such as OCs have been linked to increased susceptibility to cardiometabolic diseases and an elevated risk of myocardial infarction. The gut microbiota is thought to be a critical regulator of cardiometabolic disease risk; however, its interactions with OC use remain understudied. **OBJECTIVE:** We aimed to evaluate the effects of OC use on the intestinal microbiota and investigate microbial associations with intestinal estradiol levels, energy homeostasis, and hepatic oxidative stress markers. **METHODS:** Female C57BL/6J mice were fed a high-fat diet with or without OCs from 7 to 8 weeks of age and maintained for either 12 or 20 weeks. Duodenal, jejunal, cecal, and colonic microbiota, cecal short- and branched-chain fatty acids, and intestinal estradiol levels were assessed. **RESULTS:** Both 12- and 20-week of OC treatments significantly elevated colonic estradiol levels. Twenty weeks of OC treatment significantly altered the composition of both cecal and colonic microbiota and increased cecal isobutyric acid concentrations, whereas 12 weeks of OC treatment resulted in only trending shifts in the cecal microbiota and did not alter colonic microbiota or fatty acid compositions assessed. In 12-week treated mice, cecal *Lactococcus* was positively associated with non-resting energy expenditure, whereas in 20-week treated mice, cecal *Lachnoclostridium* was positively associated with resting energy expenditure. **CONCLUSIONS:** OC use induces time- and intestinal segment-dependent shifts in the gut microbiota and branched-chain fatty acid production. The OC-induced increase in colonic estradiol could further influence the gut microbiota and health when utilized long-term. These findings provide critical insight into how OC use may contribute to increased cardiometabolic risk through gut microbial alterations.

## 1. Introduction

The gut microbiota comprises trillions of microbes and plays a crucial role in maintaining host health, influencing factors such as adiposity, cardiovascular disease, and sex hormone regulation [1,2]. This complex microbial community is highly dynamic and can be easily shaped by both internal physiological systems and external factors, including diet and medications. Recent research has demonstrated that fluctuations in circulating estrogen levels can significantly alter the gut microbiota [3]. Conversely, germ-free mice, lacking a naturally occurring microbiota, have altered estrus cycles, which can be normalized by the introduction of microbes [4]. The gut microbiota has also been shown to utilize androgens, including testosterone, as carbon sources, thereby impacting circulating hormone levels [5]. This evidence indicates a bidirectional regulatory relationship between sex steroids and the gut microbiota.

Estrogen is generally considered to have beneficial effects, with both the hormone and its receptors playing key roles in anti-inflammatory and cardio-protective benefits [6,7]. Evidence suggests that menstruating premenopausal women with higher circulating estrogen levels have a reduced risk of cardiometabolic diseases, such as type 2 diabetes [8]. In contrast to this evidence of the protective effects of endogenously synthesized estrogen, the use of synthetic estrogen as contraception in reproductive-age women and as supplementation in post-menopausal women have both been shown to induce unfavorable cardiometabolic risks. For example, the use of combined oral contraceptives (OC) containing estrogen is associated with increased risk of cardiometabolic incidence and incidence of stroke [8,9,10,11]. Additionally, OCs have been shown to induce hyperinsulinemia in mice [12]. Our research team has previously reported that OC treatment in high-fat-diet fed mice resulted in increased metabolic stress largely associated with increased markers of hepatic oxidative stress and production of reactive oxygen species [13]. Importantly, cardiometabolic disease risk and progression are thought to be strongly influenced by the gut microbiota and their collective metabolic output [14,15]. Though research is limited, human studies have suggested that the gut microbiota is impacted by OC use, with women using OCs containing etonogestrel, a synthetic estrogen, exhibiting an increased relative abundance of *Erysipelatoclostridium* [16]. Additionally, circulating estradiol and sex hormone-binding globulin in women using OCs were found to be positively associated with *Eubacterium ramulus* [17].

To date, limited research has explored the effects of OC use on the gut microbiota, and no studies have investigated the potential connection between OC-induced metabolic stress and microbial composition. Approximately 65% of reproductive-aged women in the United States use some form of contraception, with OCs being the second most utilized form [18]. While most women use OCs to prevent pregnancies, OCs are also commonly prescribed for non-contraceptive reasons, such as management of fibroids, menstrual pain, and irregular menstruation [19,20]. Thus, understanding the role of OCs in shaping the gut microbiome may be critical to optimize women’s health. Herein, we aimed to evaluate the direct effects of two different durations of OC treatment on the gut microbiota in mice. Because women may use oral contraceptives for varying durations, we employed two treatment lengths in this study to assess potential effect of treatment duration. Additionally, we examined the relationships between the gut microbiota, intestinal estradiol, and hepatic markers of metabolic stress.

## 2. Methods

### 2.1. Ethical Approval

The animal protocol was approved by the Institutional Animal Care and Use Committee at the University of Kansas Medical Center (Protocol #2019-2539). All mice were sacrificed using methods approved for humane euthanasia for the species, stage of development, and size. Mice were anesthetized with phenobarbital (0.5 mg/g BW) via intraperitoneal injection before terminal procedures.

### 2.2. Experimental Design

Our experiment performed follow-up analysis on two main cohorts: 12-week and 20-week treatment lengths previously published (*n* = 12/group for 12-week and *n* = 8/group for 20-week) [13]. These sample sizes were chosen based on previous work showing their adequacy for detecting hormonal-driven shifts in the gut microbiome [21]. Briefly, five- to six-week-old female C57Bl/6J mice (Jackson Laboratories, Bar Harbor, ME, USA) were individually housed with plastic huts and cotton nestlet enrichment at 30 °C on a reverse light cycle (light 22:00–10:00). Mice were housed at 30 °C, which is within the thermoneutral range for rodents, to limit excess energy expenditure necessary to regulate body temperature [22]. Mice had ad libitum access to water and a high-fat diet (HFD; D12451: 45% kcal fat, 17% kcal sucrose; 4.7 kcal/g; Research Diets, New Brunswick, NJ, USA) with or without the addition of OC, which includes 2 mg ethinylestradiol (EE) and 200 mg levonorgestrel (LNG) per kg diet. The dosage of EE and LNG in the diet was chosen based on cessation of the estrous cycle [13]. A combination of EE and LNG were chosen to mimic combined oral contraceptives, which are a commonly prescribed form in humans [23]. Further, a low-fat diet control was not included in the present study in order to assess the effects of OC in the context of metabolic stress. Before terminal procedures, mice were fasted for 2 h in the light cycle (07:00–09:00).

**Figure 1:**
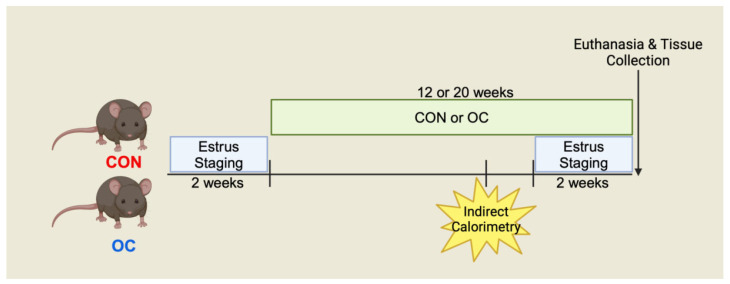
Study Schema. Female C57BL/6J mice were evaluated for estrus cyclicity at 5–6 weeks of age, then initiated on a 45% high-fat diet with supplementation of oral contraceptives (OC) or without (CON) at 7 to 8 weeks of age. This age was chosen to mimic the initiation of contraceptive use at sexual maturity. Mice were maintained on this diet for either 12 or 20 weeks (*n* = 12/group for 12-week treatment and *n* = 8/group for 20-week treatment). Indirect calorimetry was performed during weeks 9–10 or 17–18 of treatment for 12-week or 20-week cohorts, respectively. Additional estrus staging was performed for the last two weeks of the treatment period prior to euthanasia to assess the efficacy of OCs at suppressing cyclicity.

Estrus cycles were assessed for 2 weeks via vaginal cytology to determine cyclicity. Following this period, dietary OC treatment was initiated as either a high-fat diet only (CON group) or a high-fat diet + OC (OC group). Mice were 5–6 weeks old at the beginning of the experiment, 7–8 weeks of age when OC treatment was initiated, and 19–20 weeks and 27–28 weeks of age after the 12-week and 20-week interventions, respectively. Mice started the OC treatment shortly after the onset of sexual maturity to mimic the initial use of OC in humans (Figure 1).

Energy metabolism was assessed by indirect calorimetry during weeks 9–10 or 17–18 of treatment for 12-week or 20-week cohorts, respectively. The Promethion Core metabolic monitoring system (Sable Systems International, Las Vegas, NV, USA) with an environmental control cabinet programmed to 30 °C was used for these outcomes. Briefly, mice were acclimated to the indirect calorimetry cages for 3 days prior to the start of the data collection, which occurred for 5 days. Resting energy, non-resting energy, total meters, total breaks, and respiratory quotient were determined. Previously published energy expenditure data were incorporated into the present study to examine correlations with gut microbial features [24].

### 2.3. Estrous Cycle Staging

The estrous cycle stage was monitored by cytological vaginal lavage in order to ensure efficacy of the OC effect on cyclicity. Briefly, a plastic pipette filter tip was used with a latex bulb to gently flush the vagina with sterile saline 3–5 times. The sample from the final flush was expelled on a glass slide and set aside to dry. Once dry, the sample was stained with 0.1% crystal violet, rinsed with water, and a drop of glycerol was placed on top of the dried stain followed by a cover glass. Slides were observed under a light microscope and the estrous cycle stage was determined by the proportion of nucleated epithelial cells, cornified squamous epithelial cells, and leucocytes [25].

### 2.4. mRNA Gene Expression

Gene expression data used to correlate with gut microbiota data in this current report has been previously published with a detailed method [24]. Briefly, 20–25 mg of flash-frozen or freeze-clamped liver tissue was used for RNA extraction via RNeasy Mini Kit (Qiagen Sciences, Inc, Germantown, MD, USA) per the manufacturer’s protocol. cDNA was prepared using the ImProm-II reverse transcription system (Promega, Madison, WI, USA). RT-PCR was run on QuantStudio 3 (Thermo Fisher Scientific, Waltham, MA, USA) using SYBR green (Sigma Aldrich, St. Louis, MO, USA). The delta-delta Ct method was used to calculate relative gene expression, which was normalized to the housekeeping gene peptidylprolyl isomerase B (PPIB). Primer sequences are reported in Table 1. Data obtained from the CON mice were used as the reference group within each cohort.

### 2.5. Cecal Short and Branched-Chain Fatty Acids

Short and branched-chain fatty acids were quantified in the cecum using a method previously described [26]. In brief, ceca were weighed and homogenized with 1.2 g of zirconia-silicate beads using a bullet blender (Next Advance, New York, NY, USA) in 0.5% phosphoric acid. The supernatant was then mixed with an equal volume of ethyl acetate containing 0.14 mL heptanoic acid/L as an internal standard. This was vortexed for 5 min before centrifugation at. 17,000× *g* at 4 °C for 10 min. The ethyl acetate phase was recovered and stored at −80 °C until analyzed using gas chromatography (Agilent 7890A gas chromatograph, GC-FID 7890A, Santa Clara, CA, USA). Peak areas were recorded, corrected for extraction efficiency and sample volume variability using the internal standard heptanoic acid, and quantified using a standard curve.

### 2.6. Intestinal Estradiol

Intestinal estradiol concentration was determined to assess the local intestinal concentration of estradiol that may impact the gut microbiota. In brief, duodenal, jejunal, and proximal colonic tissues were homogenized in 100 µL of PBS and 40 µL of 0.1 mol/L HCl. Homogenate was then mixed with 260 µL ethyl acetate and isopropanol (mixed 1:1 volume) and centrifuged at 2000× *g* for 5 min. The ethyl acetate phase was transferred to a new tube and ethyl acetate was evaporated before resuspending in zero standard steroid-free serum [27,28], Estradiol was assessed using Sensitive Estradiol ELISA (DRG International, Marburg, Germany).

### 2.7. Genomic DNA Extraction

For cecal microbiota, genomic DNA was extracted from cecal content using the QIAmp PowerFecal Pro kit (Qiagen Sciences, Inc, Germantown, MD, USA) per the manufacturer’s protocol and eluted in 100 µL of provided elution buffer. For small intestinal and colonic microbiota, genomic DNA was extracted from scrapings of duodenum, jejunum, and proximal colon lumen. Briefly, the resected intestines were sectioned and cut open longitudinally. The content and mucosa of these intestinal segments were scraped using a sterile glass slide to account for both luminal- and mucosal-associated microbiota. QIAmp PowerFecal Pro kit was used to extract genomic DNA from intestinal scrapings and DNA was eluted in 50 µL of provided elution buffer.

### 2.8. 16S rRNA Sequencing and Analysis

Amplification of the 16S rRNA gene was performed via PCR using unique barcodes attached to forward and reverse universal primers flanking the hypervariable 4 (V4) region of this gene [29,30]. PCR was performed using KAPA HiFi HotStart DNA polymerase (KAPA Biosystems, Wilmington, MA, USA) under the following conditions: denature for 3 min at 95 °C, followed by 25 cycles of denaturation for 30 s at 95 °C, annealing for 30 s at 55 °C and elongation for 30 s at 72 °C, and a final elongation step for 5 min at 72 °C. PCR products were purified using the QIAquick 96-well PCR Purification Kit (Qiagen Sciences, Inc, Germantown, MD, USA) and subsequently quantified by Invitrogen Qubit Flex Fluorometer (Invitrogen, Carlsbad, CA, USA) using the dsDNA HS Assay Kit (ThermoFisher Scientific, Waltham, MA, USA). For small intestinal samples, the following modifications were made to the procedure: (1) bovine serum albumin (BSA) was added to the PCR reaction at a final concentration of 0.1 µg/µL reaction volume and (2) the PCR cycle was increased to 30 to account for low biomass present compared to cecum (3) PCR products were purified using band selection on low melt agarose gel (Fisher Scientific, Waltham, MA, USA). DNA was extracted from the gel using a gel DNA recovery kit per the manufacturer’s protocol (Zymo Research, Irvine, CA, USA). DNA concentration was quantified by Invitrogen Qubit Flex Fluorometer (Invitrogen, Carlsbad, CA, USA) using the dsDNA HS Assay Kit (ThermoFisher Scientific, Waltham, MA, USA). These barcoded libraries were then pooled in an equimolar ratio and sequenced at the Bindley Bioscience Center at Purdue University using v2 chemistry of the Illumina MiSeq platform (Illumina, San Diego, CA, USA) to generate 2 × 250 bp pair-end reads.

Sequences were processed using Quantitative Insights into Microbial Ecology (QIIME) 2 pipeline (2023.5) [31]. Briefly, demultiplexed paired-end sequences were imported using Casava 1.8 format and denoised using DADA2 to obtain an amplicon sequence variant (ASV) table [32]. ASV present less than four times per sample or present in less than 2 samples were discarded. A naive Bayes taxonomy classifier was trained on the SILVA reference database (clustered at 99% similarity) [33]. This classifier was used to assign taxonomy to ASVs [34]. Alpha diversity was assessed via Shannon Indices. Beta diversity was assessed using principal coordinates analysis (PCoA) of unweighted UniFrac, weighted UniFrac, Bray–Curtis, and Jaccard distance metrics. Distances between treatment groups were tested by PERMANOVA using QIIME2 [35]. The relative abundance of taxa was not assumed to follow a normal distribution; therefore, differential abundance was assessed using Mann–Whitney U for comparisons between two groups or Kruskal–Wallis with Dunn’s post hoc correction for comparing all four groups.

### 2.9. Statistical Analysis

To visualize taxa affected by dietary OC and their importance within the microbial community, co-occurrence networks were constructed. To construct the co-occurrence networks, a Spearman correlation matrix (edges) and importances were then assigned to nodes (taxa) via a custom script in Python version 3.12.10, and graphics were created in Cytoscape version 3.10.2 [36,37,38]. The importance of edge connections was set as the absolute sum of correlation coefficients (ρ). Only significant edge connections (*p* < 0.05) were included in the network, and nodes were locationally fixed across all networks to improve visual assessment of OC-induced changes.

Spearman correlations were performed between colonic estradiol levels and identified genus in the colon, in addition to cecal genus and hepatic gene expression. Cecal short- and branched-chain fatty acids, and intestinal estradiol data were assessed for outliers using robust regression and outlier removal (ROUT). Normality was assessed using the Shapiro–Wilk test. Variables found to be normally distributed were assessed using an unpaired *t*-test or one-way analysis of variance (ANOVA). Variables that were not normally distributed were assessed with Mann–Whitney U or Kruskal–Wallis with Dunn’s post hoc analysis. Differential abundance analysis was performed under the assumption of non-normal distributions and was assessed with Mann–Whitney U or Kruskal–Wallis with Dunn’s post hoc analysis. Data analysis was performed in R version 4.4.3 or Python and graphs were generated using Prism version 9.4.1 (GraphPad Software, San Diego, CA, USA) [37,38]. Statistical significance was set at *p* < 0.05, and trending differences were considered at *p* < 0.1.

## 3. Results

### 3.1. Oral Contraceptive Usage Impacted Metabolic State in Mice

**Figure 2:**
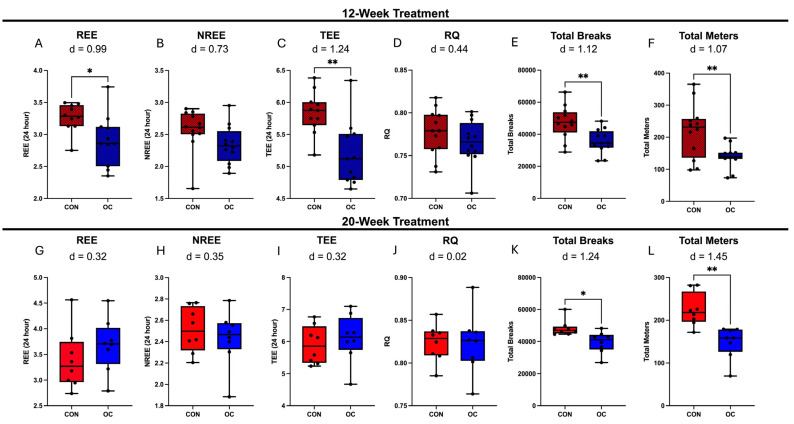
Indirect calorimetry was utilized to determine the effect of 12- or 20-week OC treatment on metabolic parameters (*n* = 7–12/group). 12-week OC treatment resulted in significantly reduced resting energy expenditure (REE) and total energy expenditure (TEE) but did not impact non-resting energy expenditure (NREE) or respiratory quotient (RQ) (**A**–**D**). A total of 12 weeks of OC treatment also resulted in reduced total breaks and total meters (**E**,**F**). A total of 20 weeks of OC treatment did result in alterations in REE, NREE, TEE, or RQ (**G**–**J**); however, a reduction in total breaks and total meters was observed (**K**,**L**). The data presented in this figure were re-analyzed from our previously published study. Effect size shown as Cohen’s D (d). * *p* < 0.05, ** *p* < 0.01.

Our research team previously reported the effect of OCs on metabolic health in mice fed a high-fat diet [24]. Indirect calorimetry was performed at weeks 9–10 or 17–18 of treatment for 12-week or 20-week cohorts, respectively. To aid in the interpretation of the current findings, we include some of the previously published metabolic health measurements from relevant groups. These data are presented solely for contextual reference. Compared to CON, 9-10 weeks of OC treatment reduced (*p* < 0.05) resting energy expenditure (REE) and total energy expenditure (TEE) with large effect sizes of 0.99 and 1.24, respectively. However, no difference was observed in non-resting energy expenditure (NREE) (Figure 2A–C). Further, 9–10 weeks of OC treatment did not significantly impact respiratory quotient (RQ) but did induce reduced (*p* < 0.05) physical activity as measured by total breaks and total meters with large effect sizes of 1.12 and 1.07, respectively (Figure 2D–F). In 17-18 weeks of OC treatment, no difference was noted in REE, NREE, TEE, or RQ (Figure 2G–J). However, OC significantly lowered (*p* < 0.05) total breaks and meters traveled with large effect sizes of 1.24 and 1.45.

**Figure 3:**
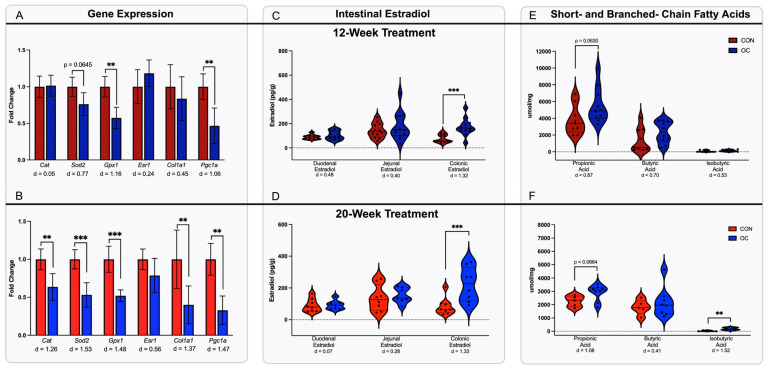
Hepatic gene expression was determined for markers of oxidative stress, fatty liver disease, and fibrosis (*n* = 7–12/group). 12-week treatment did not impact Catalase (*Cat*), or estrogen receptor (*Esr1*), but did result in reduced expression of superoxide dismutase 2 (*Sod2*) and glutathione peroxidase 1 (*Gpx1*). Further, 12-week OC did not impact collagen 1a1 (*Col1A1*), but reduced expression of peroxisome proliferator-activated receptor gamma coactivator 1-alpha (*Pgc-1a*) (**A**). In the 20-week cohort, OC resulted in reduced expression of *Cat*, *Sod2*, and *Gpx1* compared to CON (**B**). No reduction was observed in *Esr1* gene expression (**B**). After 20 weeks of treatment, OC resulted in reductions in Col1A1 and *Pgc-1a*. Estradiol levels were determined in the duodenum, jejunum, and colon of mice treated with or without OCs for 12- or 20 weeks (7–12/group). OC treatment did not result in differences in estradiol levels in either the duodenum or the jejunum of 12- or 2-week treated mice (**C**,**D**). However, OCs resulted in significantly increased colonic estradiol compared to CON in both 12-week and 20-week cohorts (**C**,**D**). Short- and branched-chain fatty acids were determined in the cecal content of mice with OC or without OC for 12- or 20 weeks (*n* = 5–10/group). After 12 weeks of treatment, OC tended to increase propionic acid but did not alter butyric acid or isobutyric acid compared to CON (**E**). After 20 weeks of treatment OC resulted in trending increases in propionic acid, no differences in butyric acid, and significant increases in isobutyric acid compared to 20-week CON (**F**) Gene expression data presented in this figure are re-analyzed from a previously published study. Effect size shown as Cohen’s D (d). ** *p* < 0.01, *** *p* < 0.001.

Gene expression was performed to assess markers of hepatic oxidative stress, fatty liver disease, and fibrosis, namely, estrogen receptor alpha (*Esr1*), catalase (*Cat*), superoxide dismutase 2 (*Sod2*), glutathione peroxidase 1 (*Gpx1*), collagen 1a1 (*Col1a1*), and peroxisome proliferator-activated receptor gamma coactivator 1-alpha (*Pgc-1a*). Markers of hepatic oxidative stress, fatty liver disease, and fibrosis were selected due to their established relevance in the metabolic response to HFD-induced metabolic dysfunction [39]. In 12-week treated mice, OC treatment did not impact *Cat* but tended to reduce (*p* = 0.0645) expression of *Sod2* with a moderate effect size of 0.77 and significantly reduced expression of *Gpx1* (Figure 3A) 12-week OC treatment also induced significantly lower expression of *Pgc-1a*, but did not alter expression of *Esr11* or *Col1a1* (Figure 3A). In the 20-week cohort, OC treatment also significantly decreased (*p* < 0.05) *Cat*, *Sod2*, *Gpx1*, *Col1a1*, and *Pcg1a*, but did not alter *Esr1* compared to CON (Figure 3B). A reduction in the expression of these genes suggests that OC treatment leads to increased reactive oxygen species in the liver, impaired mitochondrial biogenesis, and decreased collagen synthesis. In the context of the observed alterations in energy expenditure, these data indicate that OC usage may be detrimental to metabolic health.

### 3.2. Oral Contraceptive Usage Increased Concentrations of Estradiol in the Colon

Given our previous findings that oral contraceptives influence both energy expenditure and hepatic oxidative stress, we sought to investigate whether local estrogen levels may also play a role in modulating the intestinal microbiota [24]. In this study, estradiol levels were measured in the proximal small intestine (duodenum and jejunum) and proximal colon and correlated with the composition of the intestinal microbiota within each segment. Understanding this connection may further elucidate mechanisms by which estradiol influences host metabolic health. Estradiol levels in the duodenum and jejunum were not different in either treatment cohort compared to their respective controls (Figure 3C,D). However, in the colon, OC treatment resulted in significantly greater (*p* < 0.05) estradiol levels compared to CON in both treatment durations (Figure 3C,D). Differences observed were accompanied by large effect sizes of 1.32 for the 12-week colonic estradiol and 1.33 for the 20-week colonic estradiol. These results suggest a strong biological effect of OC treatment on local levels of estradiol in the colon.

### 3.3. Oral Contraceptive Usage Differentially Impacted Cecal Microbial Short- and Branched-Chain Fatty Acids

Short- and branched-chain fatty acids—including propionic, butyric, and isobutyric acid—were measured in cecal contents to assess changes in bacterial metabolites relevant to host health [40,41]. To the best of our knowledge, the impact of OCs on these fatty acids has not yet been reported. Notably, short-chain fatty acids such as butyrate have demonstrated protective effects against oxidative stress and diet-induced hepatic fibrosis. Thus, characterizing the effects of OCs on gut microbial metabolic output may help clarify the role of the microbiome in OC-induced hepatic oxidative stress. In the 12-week treatment group, OC tended to increase (*p* = 0.063) cecal propionic acid concentration with an effect size of 0.87 (Figure 3E). However, no differences were noted in cecal butyric or isobutyric acid levels (Figure 3E). Similarly, 20 weeks of OC treatment tended to increase (*p* = 0.066) propionic acid, but not butyric acid (Figure 3F). The effect size of propionic acid was 1.08, indicating a strong biological effect. However, 20 weeks of OC treatment resulted in significantly higher (*p* < 0.05) concentrations of the branched-chain fatty acid isobutyric acid compared to CON (Figure 3F).

### 3.4. Oral Contraceptive Usage Altered Intestinal Microbiota Alpha- and Beta-Diversity in a Site-Specific Manner

**Figure 4:**
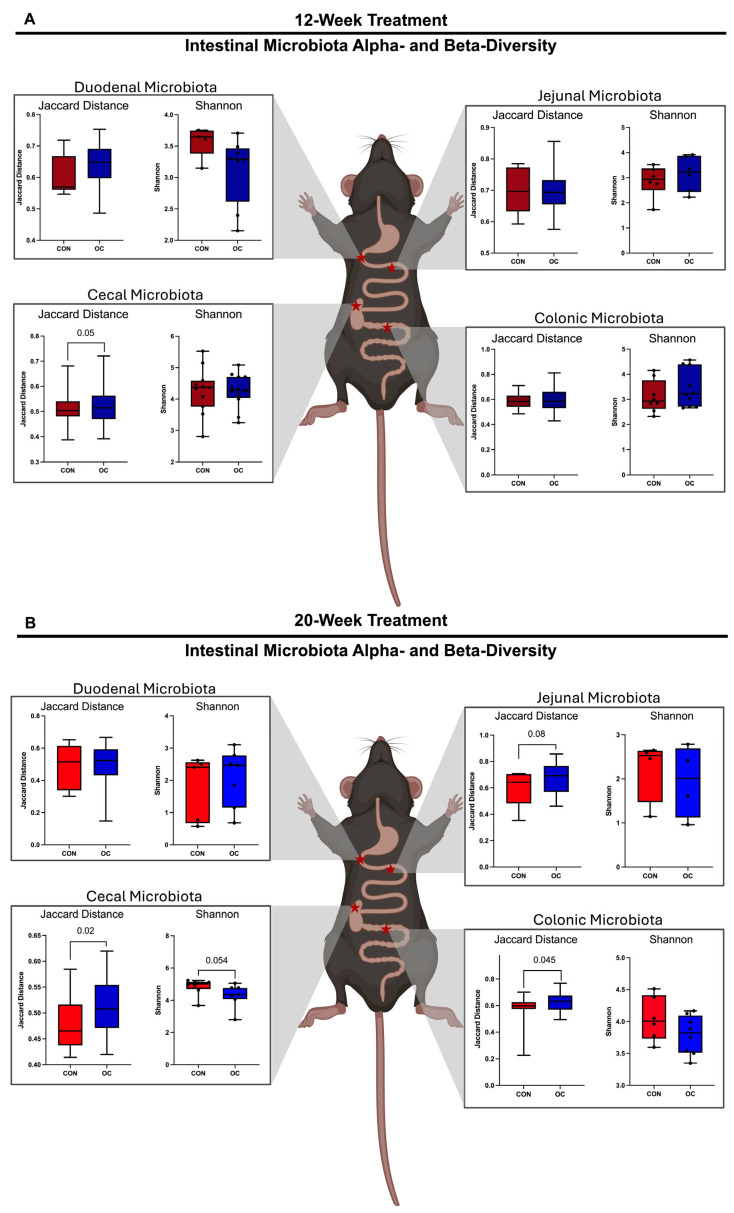
Duodenal: jejunal, cecal, and colonic microbiota were assessed after 12 or 20 weeks with or without OCs (*n* = 4–12/group). Assessment of beta-diversity metrics showed that 12 weeks of OCs did not alter duodenal or jejunal microbiota but tended to shift cecal microbiota (*p* = 0.050). After 12 weeks of treatment, OCs did not impact colonic microbiota (**A**). After 20 weeks of OC treatment, no effect was observed in duodenal microbiota, but significant community shifts were noted in jejunal, cecal, and colonic microbiota (**B**). After 12 weeks of treatment, OC did not result in alterations of alpha diversity in any intestinal segments. After 20 weeks of treatment did not alter alpha-diversity in small intestinal microbiota but tended to decrease (*p* = 0.054) alpha-diversity in cecal microbiota. No differences in alpha-diversity in the colonic microbiota were noted.

Microbiota was assessed via 16S rRNA gene sequencing in the content of duodenum, jejunum, cecum, and colon to measure segment-specific effects of OC treatment on intestinal microbiota. In the 12-week group, no differences in beta diversity were observed in duodenum or jejunum microbiota (Figure 4A and Figure S1). OC treatment tended to induce (*p* = 0.050) alterations of the cecal microbiota but did not impact colonic microbiota compared to CON (Figure 4A,B and Figure S1). In the 20-week group, no differences were observed in duodenal microbiota beta-diversity; however, trending shifts were observed in jejunal microbiota (*p* = 0.08) (Figure 4B and Figure S1). Further, OC induced significant shifts in the cecal (*p* = 0.002) and colonic (*p* = 0.045) microbiota compared to CON (Figure 4B and Figure S1).

Assessment of alpha diversity revealed that 12-week OC treatment did not induce shifts in alpha diversity in duodenal, jejunal, or colonic microbiota for either treatment duration (Figure 4A,B). However, 20-week OC treatment tended to decrease (*p* = 0.054) alpha-diversity in the cecum (Figure 4B). This evidence suggests a time-dependent shift in the cecal microbial community induced by OC usage, but not in other segments assessed.

### 3.5. Oral Contracteptive Usage Significantly Altered Proportions of Taxa in Cecal and Colonic Microbiota

**Figure 5:**
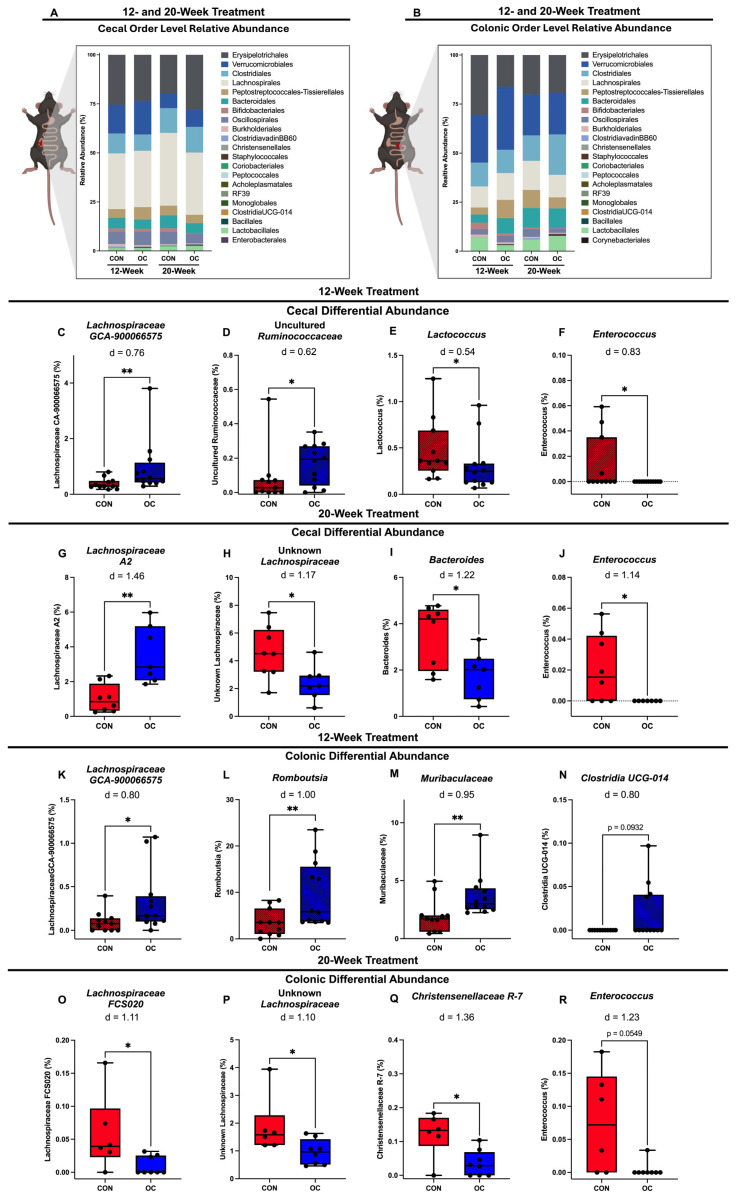
Shifts in relative abundance at the order level were also noted in both 12- and 20-week cecal and colonic microbiota (**A**,**B**) (*n* = 7–12/group). In the cecal microbiota, 12-week OC treatment promoted a significant increase in the proportions of *Lachnospiraceae GCA-900066575*, an uncultured taxon from the family *Ruminococcaceae*, and decreased proportions of *Lactococcus* and *Enterococcus* (**C**–**F**). After 20 weeks, OCs induced significant increases in *Lachnospiraceae A2*, and decreases in an unknown taxon from *Lachnospiraceae*, *Bacteroides*, and *Enterococcus* (**G**–**J**). In the colon, 12-week OC treatment resulted in increased proportions of *Lachnospiraceae GCA-900066575*, *Romboutsia*, *Muribaculaceae*, and tended to increase *Clostridia UCG-014* compared to CON (**K**–**N**). The 20-week OC treated mice exhibited decreases in proportions of *Lachnospiraceae FCS020*, and unknown genus from the family *Lachnospiraceae*, *Christensenellaceae R-7*, and tended to decrease *Enterococcus* (*p* = 0.054) compared to CON mice (**O**–**R**). Effect size shown as Cohen’s D (d). * *p* < 0.05, ** *p* < 0.01.

Given the evidence supporting the effect of OC-usage on the cecum and colon based on colonic estradiol, short- and branched-chain fatty acids, and beta-diversity metrics, further analysis of the community structure of phylogeny at the order level was performed for the cecal and colonic microbiota. In the cecal microbiota of 20-week groups, the relative abundance of *Erysipelotrichales* is 40% higher in OC-treated group compared to CON (19.64% in CON mice and 27.97% in OC-treated mice, Figure 5A). In 12-week colonic microbiota, phylogeny at the order level showed that *Erysipelotrichales* was lowered from 30.66% in CON mice to 16.13% in OC mice. Additionally, 12-week OC mice had a greater relative abundance of *Verrucomicrobiales* compared to CON mice (OC: 32.17% vs. CON: 24.21%; Figure 5B). However, in the 20-week group, CON mice had a greater relative abundance of *Clostridiales* than CON mice (OC: 20.65% vs. CON: 12.98%; Figure 5B).

Differential abundance was also performed at the genus level in the cecal microbiota to assess specific taxa impacted by OC within each treatment duration group. In 12-week treated mice, OC induced significantly greater (*p* < 0.05) proportions of *Lachnospiraceae GCA-900066575* and an uncultured taxon from the family *Ruminococcaceae* compared to CON mice (Figure 5C,D). Further,12 weeks of OC treatment significantly lowered (*p* < 0.05) the relative abundance of *Lactococcus* and *Enterococcus* compared to CON mice (Figure 5E,F). In 20-week treated mice, OC treatment resulted in significantly greater (*p* < 0.05) proportions of *Lachnospiraceae A2* and lowered (*p* < 0.05) an unknown taxon from the *Lachnospiraceae* family, *Bacteroides*, and *Enterococcus* (Figure 5G–J). Many species from these aforementioned genera have been previously described to be either associated with circulating sex steroid levels or involved in modulating host health, highlighting the potential importance of the observed alterations in the cecal microbiota [42,43].

Differential abundance analysis of the colonic microbiota showed that in the 12-week group, OC significantly increased (*p* < 0.05) the relative abundance of *Lachnospiraceae GCA-900066575*, *Romboutsia*, and *Muribaculaceae* compared to CON mice (Figure 5K–M). Further, OC treatment tended to increase (*p* = 0.093) *Clostridia UCG-014* compared to CON (Figure 5N). In the 20-week colonic microbiota, OC significantly decreased (*p* < 0.05) the relative abundance of *Lachnospiraceae FCS020*, an unknown taxon from the family *Lachnospiraceae*, and *Christensenellaceae R-7* (Figure 5O–Q). Similarly to findings in cecal microbiota, OC treatment also tended to decrease (*p* = 0.054) the proportions of *Enterococcus* in the colon (Figure 5R).

### 3.6. Oral Contraceptive Usage Impacts Cecal and Colonic Microbiota Community Dynamics in a Time-Dependent Manner

**Figure 6:**
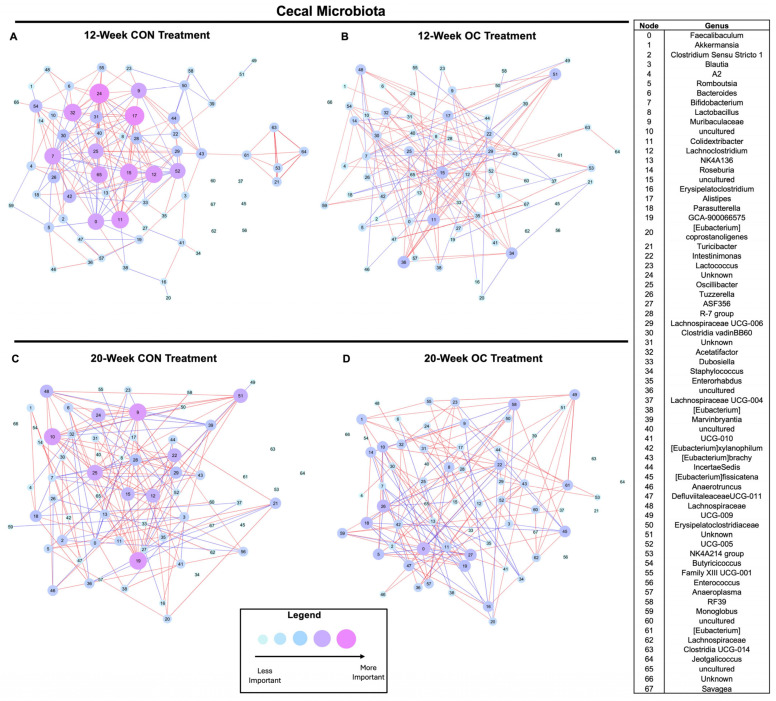
Co-occurrence networks constructed of the cecal microbiota from 12-week CON (**A**), 12-week OC (**B**), 20-week CON (**C**), and 20-week OC (**D**). Nodes were set to represent identified taxa, and edges were set as significant (*p* < 0.05) correlations, with blue connections being negative correlations and red connections being positive correlations. Nodes were set at fixed locations across all networks, and node importance was set as the absolute sum of connections of a node.

Co-occurrence networks of the cecal and colonic microbiota were constructed at the genus level, where edges were used to represent significant relationships (*p* < 0.05), and nodes were used to represent identified taxa within the community (Figure 6 and Figure S2). Nodes within the network were assigned fixed locations within the graph to visualize the changes in microbial community structure between time points and treatments. The importance of nodes (taxa) within the community structure was assigned based on the absolute sum of correlation coefficients as an indication of the strength of the relationships between taxa in the network, using node size and color [44]. In essence, the larger the node size and the darker the color, the more important a given taxon is within the community.

In the cecal microbiota, 12-week OC treatment reduced the importance of *Faecalibaculum* (node 0), *Muribaculaceae* (node 9), *Lachnoclostridium* (node 12), an uncultured *Oscillospiraceae* (node 15), *Alistipes* (node 17), and an unknown *Lachnospiraceae* (node 24) compared to 12-week CON (Figure 6A,B and Figure S2; Table S1). Conversely, the importance of an uncultured *Peptococcaceae* (node 36) increased in 12-week OC mice compared to 12-week CON mice. Further, an overall decrease in connectedness between nodes within the network was observed. In 20-week treated mice, OC reduced the importance of an uncultured *Lachnospiraceae* (node 10) and *Oscillibacter* (node 25) (Figure 6C,D and Figure S2; Table S1). In both 12- and 20-week treated mice, the network importance of *Muribaculaceae* (node 9) was reduced by OC compared to CON, demonstrating a persistent impact of OC treatment on this genus regardless of the duration of OC usage.

In addition to the impact of OC within each treatment duration, comparisons were made between 12- and 20-week groups. In 20-week treated CON mice, *Lachnospiraceae GCA-900066575* (node 19) was more important within the network compared to all other treatment networks, suggesting an age effect on this taxon that was impacted by the treatment of OC (Figure 6A–D and Figure S2; Table S1). Additionally, *RF39* (node 58) was found to be more important within the network of 20-week OC mice compared to 20-week CON, 12-week CON, and 12-week OC (Figure 6A–D and Figure S2; Table S1). Thus, OC usage may impact how aging shapes the gut microbiota. Together, this evidence highlights a unique interaction between time and OC usage on the cecal microbiota.

**Figure 7:**
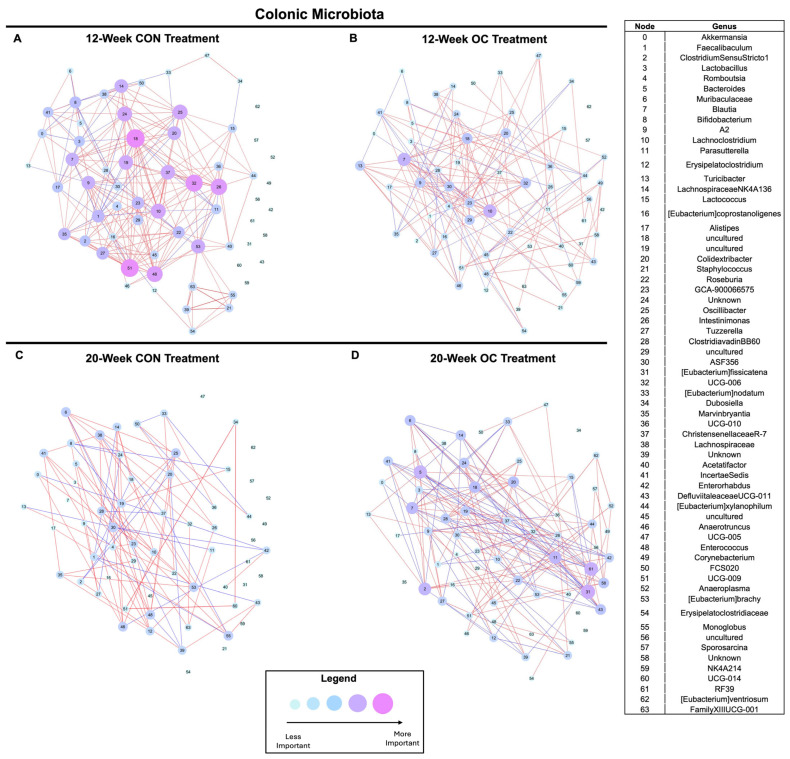
Co-occurrence networks constructed of the colonic microbiota from 12-week CON (**A**), 12-week OC (**B**), 20-week CON (**C**), and 20-week OC (**D**). Nodes were set to represent identified taxa, and edges were set as significant (*p* < 0.05) correlations, with blue connections being negative correlations and red connections being positive correlations. Nodes were set at fixed locations across all networks, and node importance was set as the absolute sum of connections of a node.

In the colon, 12-week OC treatment reduced the importance of *Lachnospiraceae NK4A136* (node 14), an uncultured *Lachnospiraceae* (node 18), an uncultured *Oscillospiraceae* (node 19), *Intestinimonas* (node 26), *Lachnospiraceae UCG-006* (node 32), *Enterococcus* (node 48), and *Butyricicoccaceae* UCG-009 (node 51) (Figure 7A,B and Figure S3; Table S2). Overall, 12-week treatment of OC resulted in reduced node weight, suggesting a strong concerted impact on the colonic microbial community structure. In 20-week treatment group, we observed the opposite effect. OC treatment resulted in increased importance of *Faecalibaculum* (node 2), *Bacteroides* (node 5), *Blautia* (node 7), *Parasutterella* (node 11), [*Eubacterium*] *fissicatena* (node 31), and *RF39* (node 61) (Figure 7C,D and Figure S3; Table S2). Further, compared to 12-week CON and OC, 20-week CON exhibited lower node importance and fewer significant connections; however, 20-week OC maintained more significant connections and higher node importance than 20-week CON. Thus, similar to the findings in cecal microbial communities, these results suggest a strong interaction between age or treatment duration and the impact of OCs on the colonic microbiota (Figure 7A–D and Figure S3; Table S2).

## 4. Colonic Estradiol Levels Are Associated with Local Bacterial Taxa Relevant to Health

Spearman correlations were performed to assess the relationship between estradiol levels and bacterial taxa locally within the colon. In 12-week treated mice, estradiol levels were positively associated with *Lachnospiraceae ASF356* (ρ = 0.612, *p* < 0.05) and an unknown *Ruminococcaceae* (ρ = 0.554, *p* < 0.05) (Figure S4A,B). Estradiol levels were also significantly negatively associated with *Bifidobacterium* (ρ = −0.458, *p* < 0.05) (Figure S4B). In 20-week treated mice, estradiol was negatively associated with *Lachnospiraceae ASF356* (ρ = −0.618, *p* < 0.05). Interestingly, this relationship was opposite to that observed in the 12-week treated group (Figure S4D). Additionally, estradiol was negatively correlated with *Lachnospiraceae FCS020* (ρ = −0.618, *p* < 0.05) and tended to be negatively correlated with *Lactococcus* (ρ = −0.511, *p* = 0.078) (Figure S4E,F).

### 4.1. Cecal and Colonic Microbial Taxa Are Associated with Physical Activity and Metabolism

Data from our previously published article suggests that OC-usage negatively impacts energy expenditure and physical activity, such as reductions in physical activity [24]. Spearman correlations were performed between cecal and colonic microbial taxa and results from indirect calorimetry in order to assess the relationship between OC-induced alterations in the gut microbiota and metabolic health. In 12-week treatment group, cecal *Lactococcus* was positively associated with non-resting energy expenditure over 24 h (NREE_24hr) (ρ = 0.695, *p* < 0.05) and total meters traveled (ρ = 0.570, *p* < 0.05). *Blautia* in the cecum was positively related to NREE_24hr (ρ = 0.558, *p* < 0.05), while *Acetatifactor* was positively associated with %NREE (ρ = 0.530, *p* < 0.05) and negatively associated with percent resting energy expenditure (%REE) (ρ = −0.530, *p* < 0.05) (Figure S5A; Table S3). In 20-week treated mice, cecal *Anaerotruncus* was positively associated with periods of inactivity (total breaks) (ρ = 0.861, *p* < 0.05), *Oscillospiraceae NK4A214* was negatively linked with total meters of movement (ρ = −0.684, *p* < 0.05), and *Lachnoclostridium* was positively associated with resting energy expenditure (ρ = 0.646, *p* < 0.05) (Figure S5B; Table S3)

In the 12-week treated colonic microbiota, *Lachnospiraceae UCG-004* was negatively linked with %REE (ρ = −0.686, *p* < 0.05) and positively linked with %NREE (ρ = 0.686, *p* < 0.05) (Figure S6A; Table S4). NREE was also associated with *Turicibacter* (ρ = 0.586, *p* < 0.05) and *Blautia* (ρ = 0.557, *p* < 0.05) (Figure S6A; Table S4). In 20-week treated colonic microbiota, *Lactococcus* was positively associated with inactivity (Total_Breaks) (ρ = 0.730, *p* < 0.05) (Figure S6B; Table S4). *Oscillibacter* was also positively related to total breaks (ρ = 0.714, *p* < 0.05), while *Lachnospiraceae FCS020* was positively associated with total meters (ρ = 0.679, *p* < 0.05) (Figure S6B; Table S4).

### 4.2. Cecal and Colonic Microbial Taxa Are Associated with Hepatic Markers of Oxidative Stress and Liver Steatosis

We assessed the relationship between gut taxa and hepatic genes related to antioxidant capacity and liver steatosis that were impacted by OC usage [24]. Specifically, OC treatment reduced gene expression of key genes necessary for H_2_O_2_ clearance and markers of mitochondrial biogenesis. In 12-week treated cecal microbiota, *Bifidobacterium* was negatively associated with *hepatic Esr1* (ρ = −0.656, *p* < 0.05) while *Alistipes* was significantly positively associated with *Esr1* (ρ = 0.646, *p* < 0.05) and *Pgc1*α (ρ = 0.700, *p* < 0.05) (Figure S7A; Table S5). Additionally, *Lachnospiraceae FCS020* was positively associated with hepatic *Cat* (ρ = 0.537, *p* < 0.05) and *Sod2* (ρ = 0.491, *p* < 0.05).

In 20-week treated mice, an uncultured *Ruminococcaceae* was positively correlated with *Cat* (ρ = 0.749, *p* < 0.05) and *Esr1* (ρ = 0.593, *p* < 0.05) (Figure S7B; Table S5). *Parasutterella* was positively associated with *Gpx1* (ρ = 0.611, *p* < 0.05), *Sod2* (ρ = 0.589, *p* < 0.05), and *Pgc1*α (ρ = 0.586, *p* < 0.05). Further, *Bacteroides* (ρ = 0.571, *p* < 0.05) and *Lactococcus* (ρ = 0.600, *p* < 0.05) were positively associated with *Sod2* gene expression (Figure S7B; Table S5).

Consistent with findings from the 12-week treated cecal microbiota, we observed that in the 12-week treated colon, *Bifidobacterium* was negatively correlated with *Esr1* (ρ = −0.613, *p* < 0.05), while *Alistipes* showed a positive correlation with *Esr1* (ρ = 0.664, *p* < 0.05) (Figure S8A; Table S6). *Tuzzerella* was negatively associated with *Sod2* (ρ = −0.543, *p* < 0.05) and *Romboutsia* was negatively associated with *Gpx1* (ρ = −0.551, *p* < 0.05). Given the similar observed relationships between cecal and colonic taxa and hepatic gene expression in 12-week treated mice (e.g., associations of *Bifidobacterium* and *Alistipes* with *Esr1),* this evidence supports conserved interactions between cecal- and colonic microbiota and hepatic gene expression.

In the 20-week colon, an uncultured taxon from the family *Peptococcaceae* was positively associated with *Esr1* (ρ = 0.757, *p* < 0.05), and *Christensenellaceae R-7* was positively related to *Cat* (ρ = 0.757, *p* < 0.05) (Figure S8B; Table S6). Further, Marvinbryantia was found to be positively linked to both *Gpx1* (ρ = 0.654, *p* < 0.05) and *Col1a1* (ρ = 0.635, *p* < 0.05) (Figure S8B; Table S6). Alterations in cecal and colonic microbiota induced by OC treatment may interact with host health through these bacterial taxa.

## 5. Discussion

In the present study, we examined the effects of two durations of OC treatment—12 and 20 weeks—on the gut microbiota in mice fed a high-fat diet. OC use altered the gut microbiota in a time- and region-dependent manner, with minimal changes after 12 weeks but significant shifts in cecal and colonic communities, including reduced cecal alpha diversity, after 20 weeks of exposure. Additionally, 20 weeks of OC treatment increased cecal isobutyric acid levels, whereas no differences were observed in the 12-week treatment group. Regardless of treatment duration, OC-treated mice had elevated estradiol levels in the colon compared to controls, but differences were not observed in duodenal or jejunal tissues. The colonic estradiol levels were associated with bacterial taxa that have been previously reported to influence host hepatic and metabolic health through their metabolites, such as short chain fatty acids [45,46,47].

*Enterococcus* was found to be significantly reduced in the cecal microbiota in both 12- and 20-week cohorts and tended to be reduced in the 20-week colonic microbiota. *Enterococcus* is a lactic acid-producing bacterium and several strains within this genus have been marketed as a probiotic due to their potential beneficial effects in the intestinal tract [48]. Specifically, supplementation of a probiotic cocktail including *Enterococcus* has been found to ameliorate high-fat diet-induced intestinal inflammation, intestinal permeability, and certain aspects of metabolic dysfunction in mice, such as glucose tolerance [49]. A clinical trial that supplemented an *Enterococcus* probiotic strain (*Enterococcus faecium M-74*) for one year decreased serum cholesterol levels in humans [50]. A six-week trial found that daily consumption of a milk product fermented with *Enterococcus faecium* lowered levels of low-density lipoprotein (LDL) in middle-aged men [51]. *Enterococcus* has also been shown to be capable of producing vitamin B_12_. Vitamin B_12_ has been inversely associated with metabolic syndrome, though the mechanism is unclear [52,53]. Thus, OC-induced reductions in *Enterococcus* proportions in the cecum may play a key role in OC-associated metabolic diseases.

In addition to reductions in *Enterococcus*, 12-week treatment of OCs resulted in reductions in *Lactococcus* in the cecum. *Lactococcus* also tended to be negatively associated with estradiol levels in the 20-week treated colon. *Lactococcus* has been shown to have anti-inflammatory effects when supplemented to a rodent model of colitis [54,55]. In a rat model of liver cirrhosis, *Lactococcus lactis* supplementation ameliorated liver damage [56]. Additionally, supplementation of *Lactococcus* bacteria attenuated non-alcoholic liver disease and atherosclerosis in rabbits [57]. In further support of the effect of OCs on liver health, reductions in the concentrations of estrogens and progestins (synthetic progesterone) in OCs have been reported to reduce the risk of adverse liver reactions in women [58]. In the present study, OC usage significantly decreases *Lactococcus* in 12-week treated mice and was associated with greater markers of hepatic oxidative stress in 20-week treated mice. We speculate that OCs may negatively impact the liver partially through reductions in the proportion of *Lactococcus* within the gut microbiota.

Determination of short- and branched-chain fatty acids revealed that OC treatment only tended to increase propionic acid; however, 20-week treatment of OC resulted in significant increases in isobutyric acid, a bacterial metabolite of amino acid fermentation, compared to control. Proteolytic fermentation and the level of intestinal isobutyric acid have been reported to increase with age [59,60]. Therefore, we postulated that the use of OC may exacerbate aging-related alterations of gut microbial metabolic processes. Higher fecal isobutyric acids have been positively associated with circulating total cholesterol and low-density lipoproteins (LDL) in humans [61]. Furthermore, isobutyric acid has also been shown to increase colorectal cancer metastasis by upregulating the expression of proteins related to cell migration in both cellular models and humans [62]. This evidence indicates that the use of OCs may promote the production of microbial metabolites that can increase the risks of various diseases.

Previous studies have suggested that estrogens present in OCs are readily absorbed in the proximal end of the small intestines [63,64]. Therefore, we expected to have observed intestinal estradiol differences in the small intestine but not the colon. Unexpectedly, we did not observe any differences in estradiol levels in the duodenum or jejunum but discovered an increased level of estradiol in the colon of OC-treated mice in both 12- and 20-week cohorts. One potential mechanism by which OCs increase colonic estradiol levels is through enterohepatic circulation. In this process, absorbed steroids enter circulation, are metabolized by the liver, and are excreted back into the intestines via bile. This allows the steroids to pass through the intestinal tract before being eliminated in the feces [65]. Steroids present in the intestinal tract are believed not only to modulate the gut microbiota by serving as carbon sources for certain taxa but also to be directly influenced by specific bacteria through processes such as deconjugation or the activity of hydroxysteroid dehydrogenases [66,67]. However, this mechanism does not explain the lack of differences in the small intestines. An alternative to enterohepatic circulation is steroid conversion pathways. Prior research has demonstrated that gut bacteria can convert certain molecules, such as glucocorticoids, into androgens [68,69,70,71]. Although, to our knowledge, no studies have specifically reported conversion to estrogens, it is plausible that bacteria could also convert C-19 steroids, such as androstenedione and testosterone, into C-18 estrogens. Consequently, OCs may influence the colonic microbial community in a way that promotes microbial steroid conversion pathways. We propose that future studies should investigate the kinetics of OCs, determining the proportion utilized systemically versus excreted into the intestinal tract, and examine how OCs impact the gut microbiota’s steroid-related enzymatic pathway activity.

As with all study and experimental designs, there are limitations to the work presented in this report. One limitation is the method used to detect estradiol in the intestines. Since steroids were extracted from whole tissue, we cannot determine whether the observed differences originate from the tissue itself or the luminal contents of the intestinal tract. Additionally, our detection method measures total estradiol without distinguishing between conjugated and unconjugated forms. Despite these constraints, our findings provide a foundation for future research to further elucidate the kinetic activity of OCs and their potential interactions with the gut microbiota that may be critical to metabolic health. Further, the present study is limited to the study of combined OCs in the context of HFD-induced metabolic disruption. This formulation of OCs was chosen based on prior literature reporting increased risk of cardiac events in women using the combination of progestins and estrogens [72]. Future studies are necessary to determine if diet may play a role in the observed OC-induced shifts in the gut microbiota and if estrogen or progestin only OCs have a differential impact on the microbial community.

## 6. Conclusions

In conclusion, we demonstrate that oral contraceptive usage significantly impacts the intestinal microbiota in a time- and intestinal segment-dependent way, with some intestinal bacterial taxa showing strong associations with the systemic effects of OCs on hepatic gene expression related to oxidative and metabolic stress. Specifically, we found that 20-week treatment with OCs had a greater impact on the gut microbiota than the 12-week treatment. Further, we report that OCs had a greater influence on the cecal and colonic microbiota that the small intestinal microbiota. While estrogens are generally recognized for their beneficial effects, exogenous sources such as OCs are linked to an elevated cardiometabolic risk. Our findings suggest that OCs may influence metabolic dysfunction partly by altering gut-associated bacteria essential for maintaining overall health. Additionally, our results reveal distinct differences in gut microbial responses based on the duration of OC use, underscoring the importance of further investigation into the consequences of long-term use of OCs. We believe that elucidating the role of oral contraceptives in shaping the gut microbiota could help inform more personalized recommendations and improve health outcomes for women who use these therapies for contraception or management of reproductive health conditions.

## Figures and Tables

**Table 1:** RT-qPCR Primer Sequences.

Gene	Forward Primer	Reverse Primer
*Cat*	CTCCATCAGGTTTCTTTCTTG	CAACAGGCAAGTTTTTGATG
*Col1a1*	AGCACGTCTGGTTTGGAGAG	ACATTAGGCGCAGGAAGGTC
*Ppargc1a*	TCACCATATTCCAGGTCAAG	TCATAGGCTTCATAGCTGTC
*Ppib*	TGGAGATGAATCTGTAGGAC	CAAATCCTTTCTCTCCTGTAG
*Sod2*	CCATTTTCTGGACAAACCTG	GACCTTGCTCCTTATTGAAG
*Esr1*	CAAGGTAAATGTGTGGAAGG	GTGTACACTCCGGAATTAAG
*Gpx1*	GGAGAATGGCAAGAATGAAG	TTCGCACTTCTCAAACAATG

## Data Availability

Sequences supporting the findings of this study can be found at https://www.ncbi.nlm.nih.gov/sra under the accession number PRJNA1242507.

## Supplementary Materials

The following supporting information can be downloaded at: https://www.mdpi.com/article/10.3390/nu17162591/s1.
