# Supplementary material for: Oral Contraceptives Induce Time- and Intestinal Segment-Dependent Shifts in the Gut Microbiota
Source: Nutrients. 2025 Aug 9;17(16):2591. doi: 10.3390/nu17162591 (PMC12388937; doi:10.3390/nu17162591)
Supplement: Supplementary file 1 [file nutrients-17-02591-s001.zip › SI Figures.pdf]

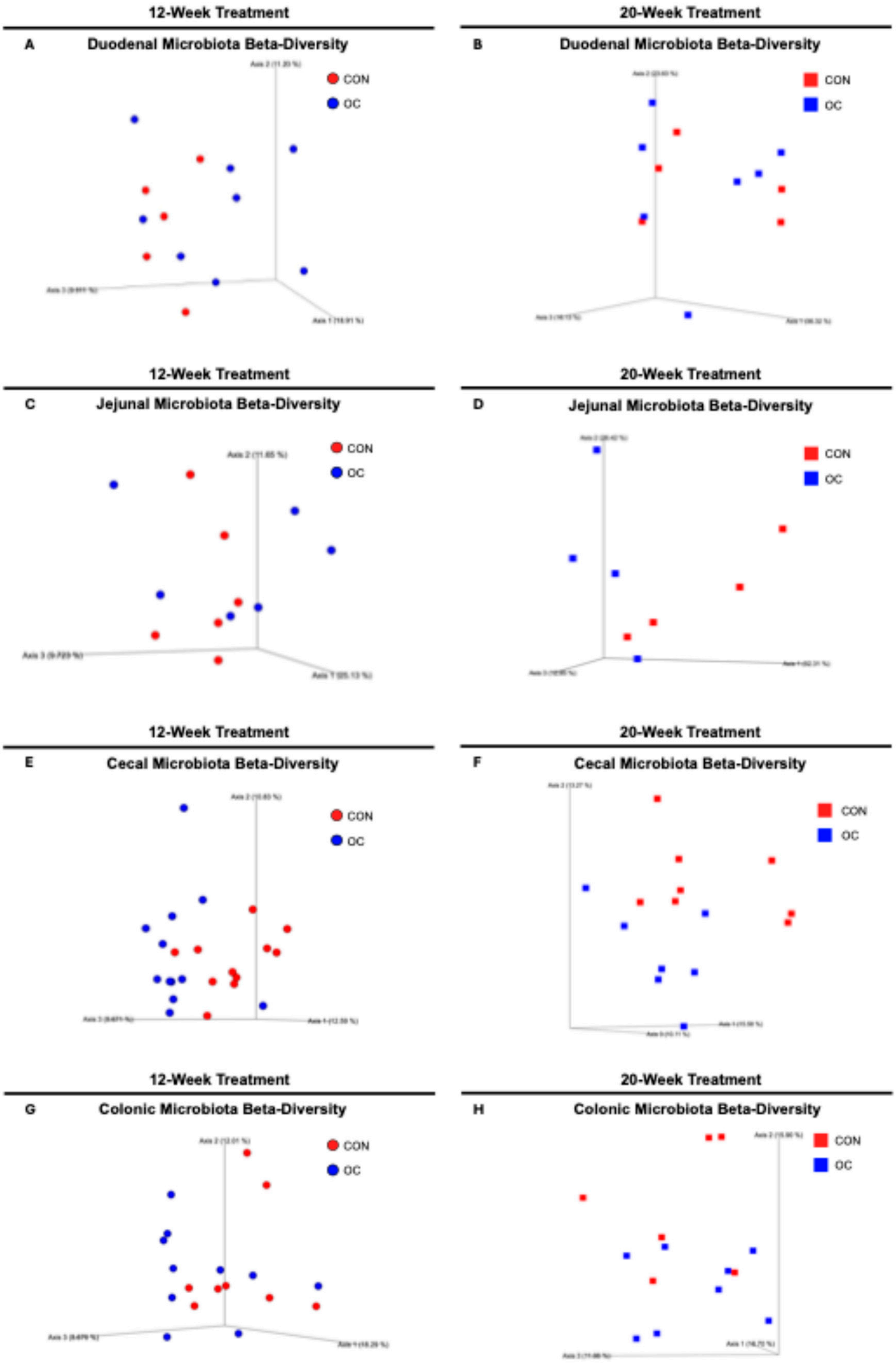

Figure S1: Principal coordinate analysis (PCoA) of Jaccard beta-diversity of 12-week and 20-week duodenal, jejunal, cecal, and colonic microbiota (A-H).

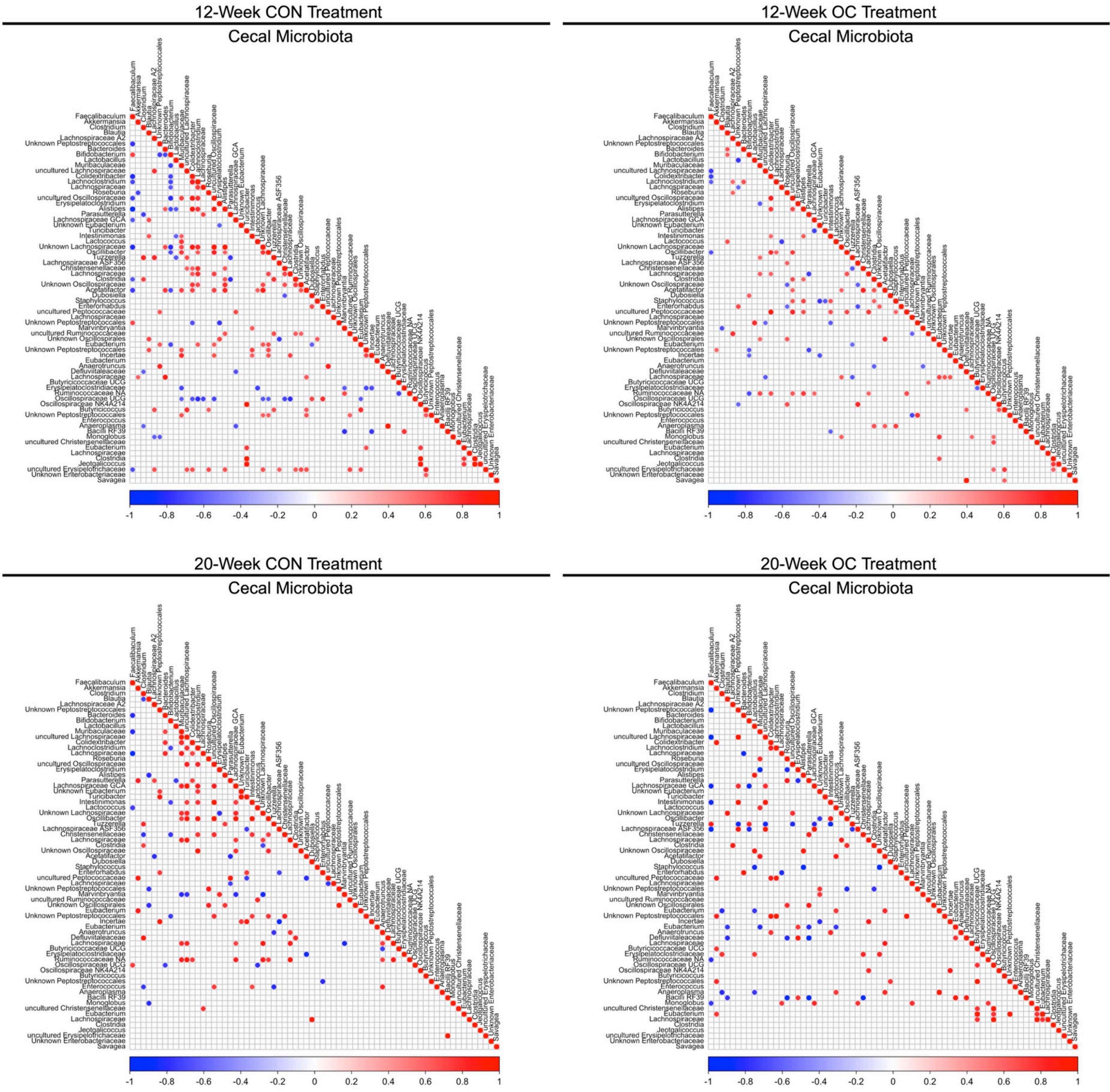

Figure S2: Heatmap of spearman correlation matrix of 12-week CON and OC cecal microbiota (A&B) and 20-week CON and OC cecal microbiota (C&D). Only significant correlations included (p<0.05).

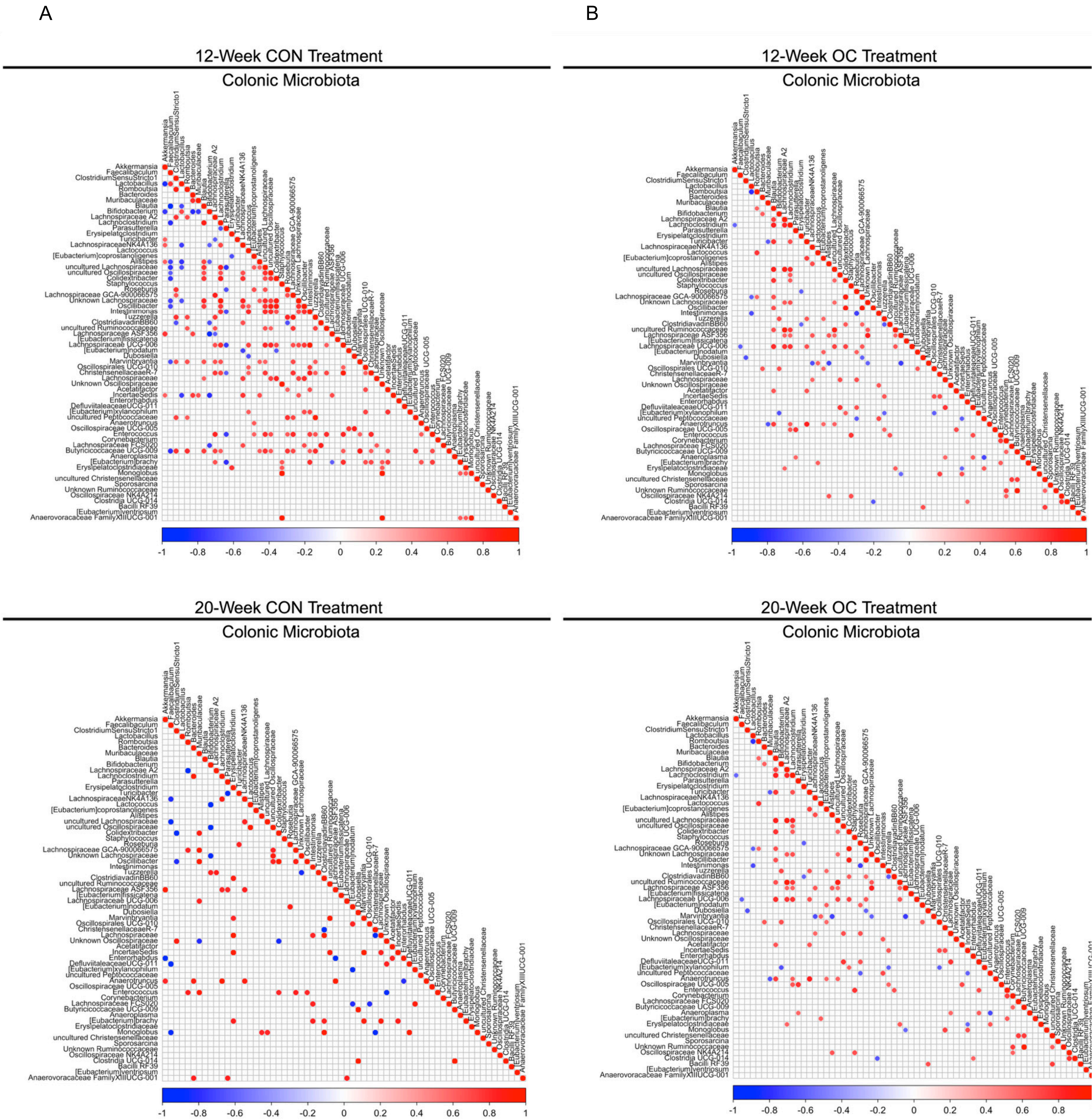

Figure S3: Heatmap of spearman correlation matrix of 12-week CON and OC colonic microbiota (A&B) and 20-week CON and OC colonic microbiota (C&D). Only significant correlations included ($p<0.05$).

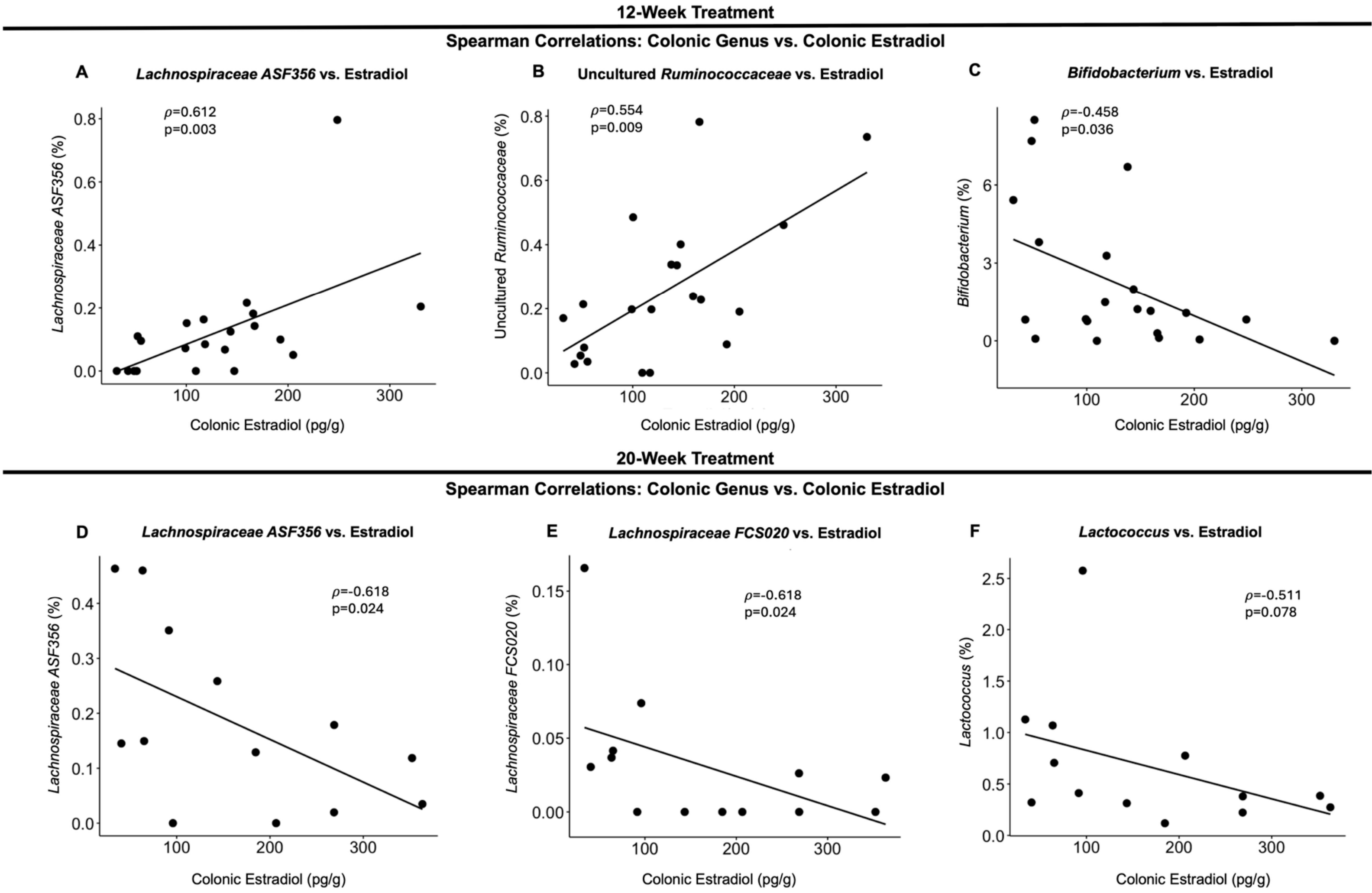

Figure S4: Spearman correlations showed that colonic estradiol levels were positively associated with *Lachnospiraceae ASF365,* and uncultured *Ruminococcaceae* in 12-week treated mice (A&B). Further, estradiol levels were negatively associated with *Bifidobacterium* in 12-week treated mice (C). In 20-week treated mice, estradiol was negatively associated with *Lachnospiraceae ASF365, Lachnospiraceae FCS020,* and tended to be negatively associated with *Lactococcus* (D-F). $^*p<0.05$, $^{**}p<0.01$, $^{***}p<0.001$

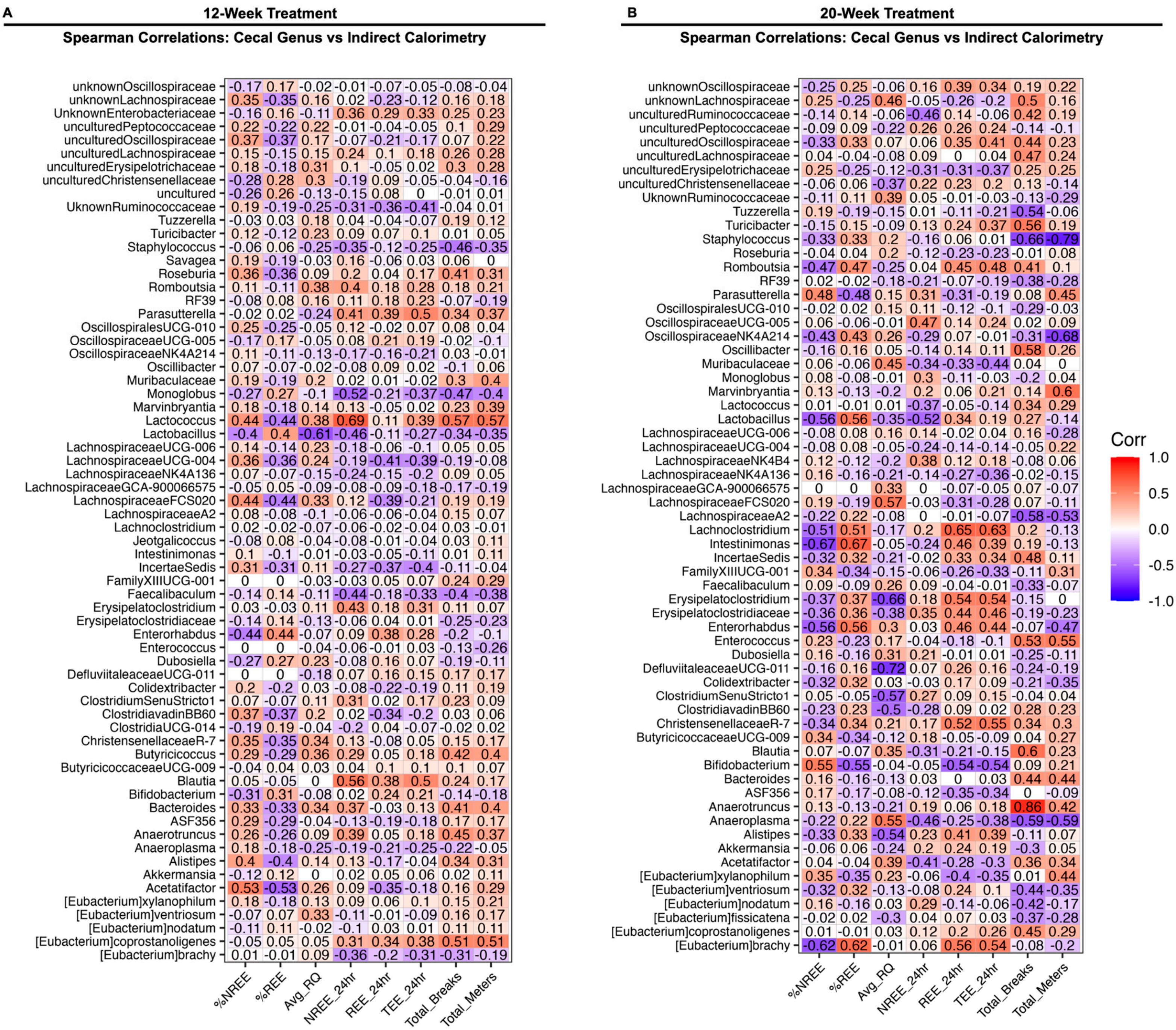

Figure S5: Spearman correlations were computed between cecal microbiota genera and metabolic parameters determined from indirect calorimetry within the 12-week (A) and 20-week (B) cohorts.

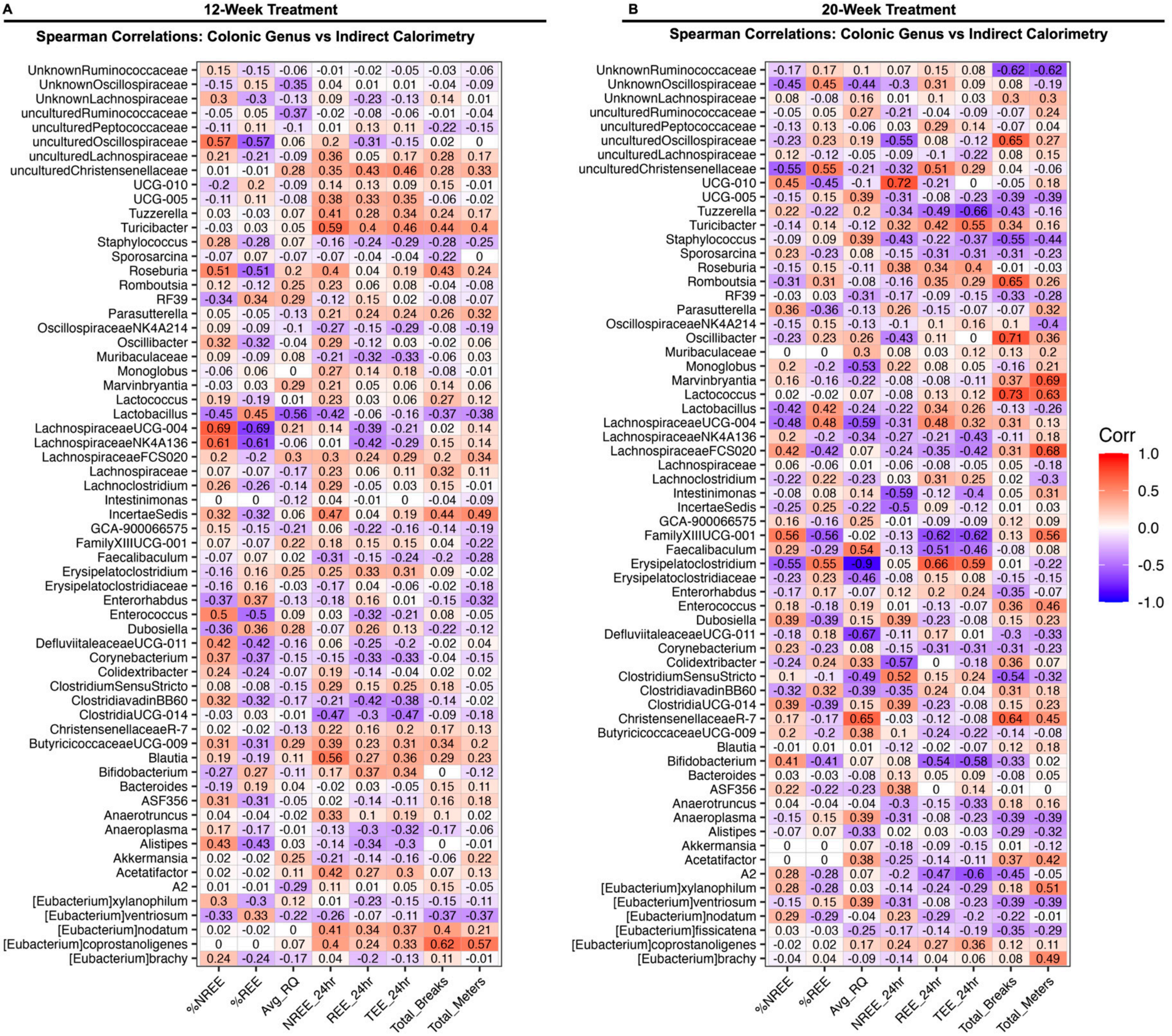

Figure S6: Spearman correlations were computed between colonic microbiota genera and metabolic parameters determined from indirect calorimetry within the 12-week (A) and 20-week (B) cohorts.

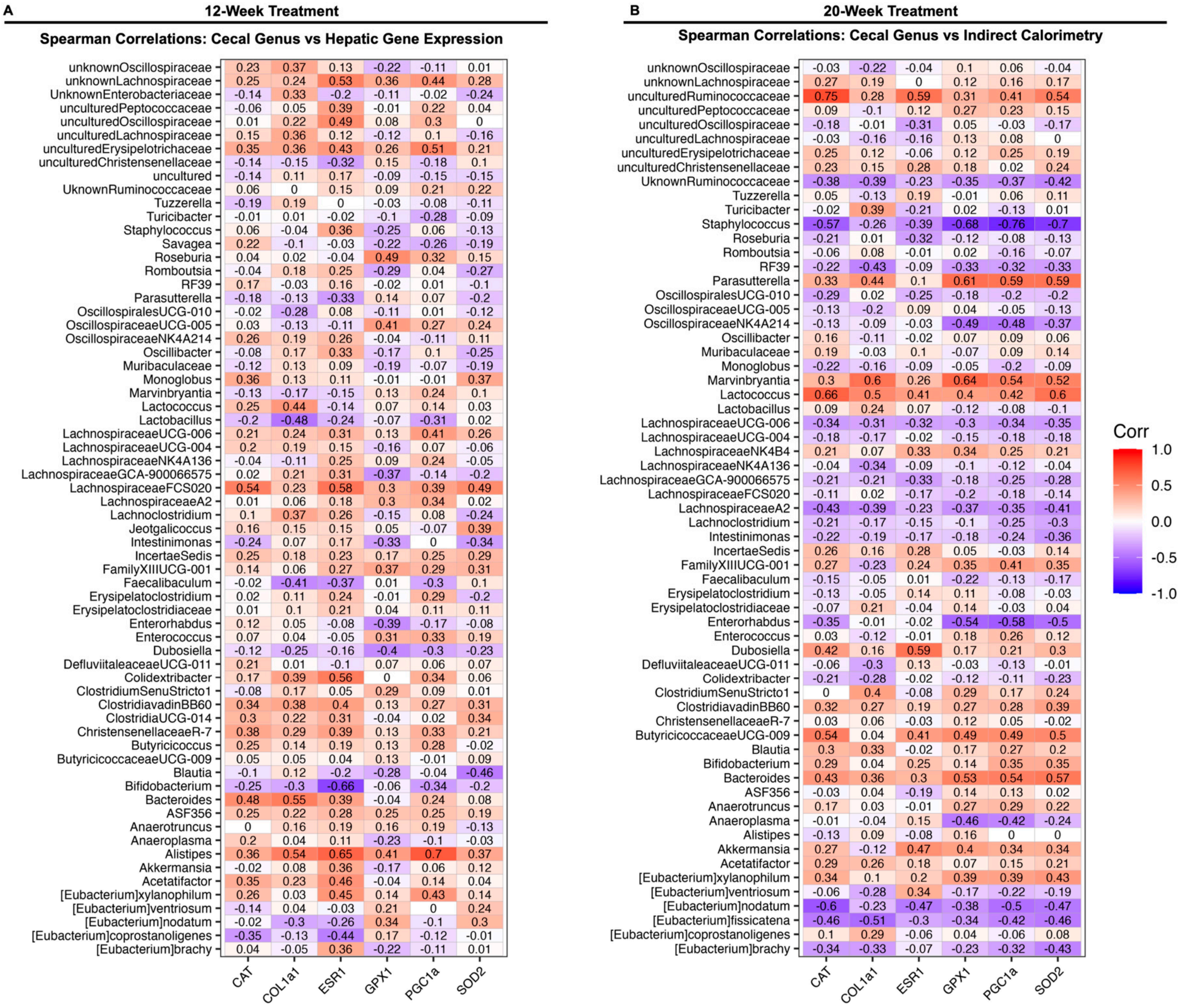

Figure S7: Spearman correlations were computed between cecal microbiota genera and hepatic gene expression of genes related to oxidative stress and metabolic health within the 12-week (A) and 20-week (B) cohorts.

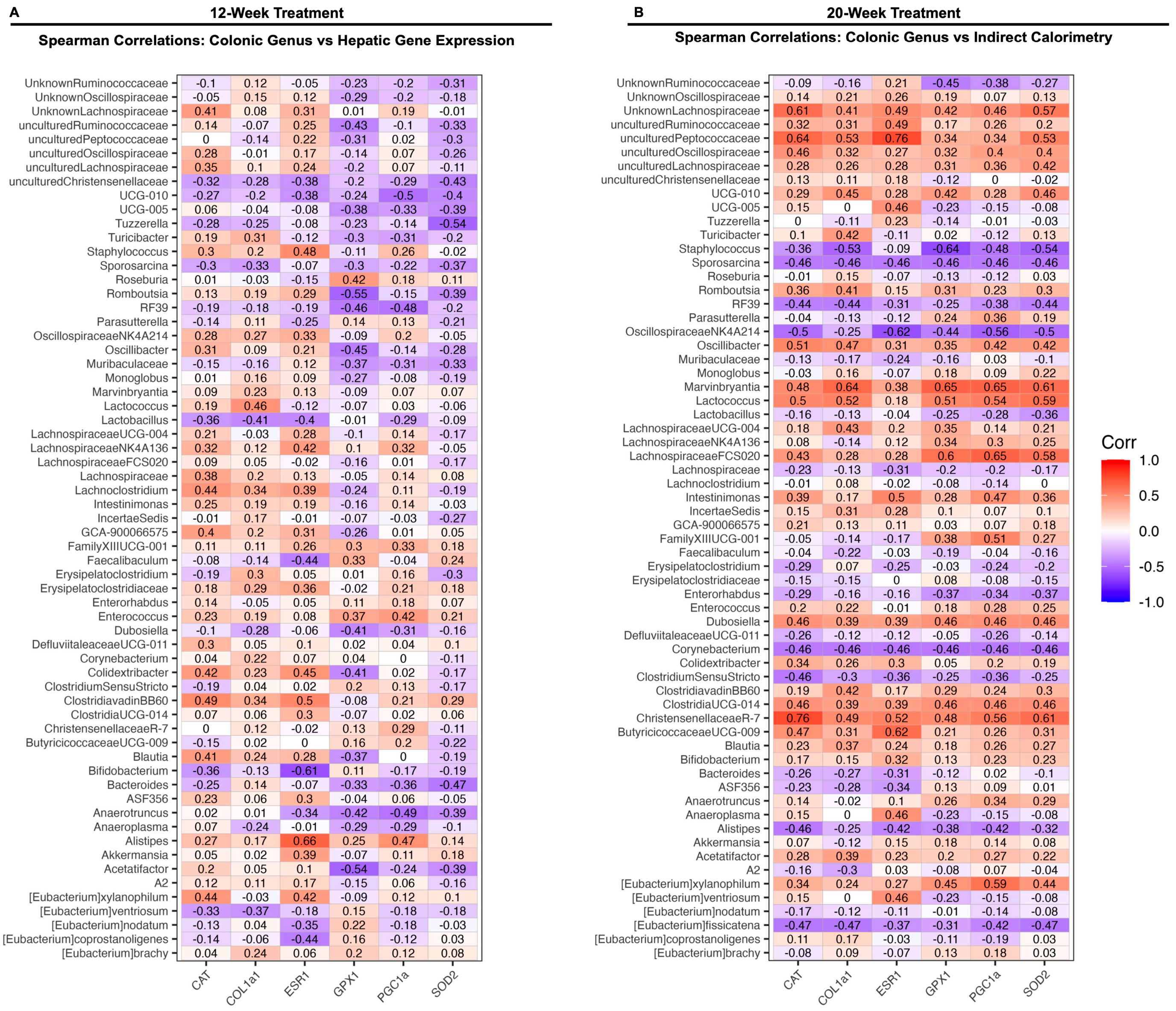

Figure S8: Spearman correlations were computed between colonic microbiota genera and hepatic gene expression of genes related to oxidative stress and metabolic health within the 12-week (A) and 20-week (B) cohorts.
